# Genomic analysis of intrahospital transmission of carbapenem-resistant Gram-negative bacteria: a multicentre study in Japan

**DOI:** 10.1099/mgen.0.001770

**Published:** 2026-07-07

**Authors:** Yasufumi Matsumura, Yohei Doi, Kayoko Hayakawa, Takehiro Hashimoto, Yusuke Tsuda, Sho Saito, Masahiro Suzuki, Aki Sakurai, Koh Shinohara, Keiichiro Mori, Kohei Uemura, Shinya Tsuzuki, Takashi Matono, Naoya Itoh, Ryota Hase, Hideaki Kato, Momoko Mawatari, Takeya Tsutsumi, Go Yamamoto, Yasuhiro Tsuchido, Masaki Yamamoto, Miki Nagao

**Affiliations:** 1 Department of Clinical Laboratory Medicine, Kyoto University Graduate School of Medicine, Kyoto, Japan; 2 Department of Microbiology, Fujita Health University School of Medicine, Aichi, Japan; 3 Department of Infectious Diseases, Fujita Health University School of Medicine, Aichi, Japan; 4 Division of Infectious Diseases, University of Pittsburgh School of Medicine, Pittsburgh, PA, USA; 5 Disease Control and Prevention Center, National Center for Global Health and Medicine, Japan Institute for Health Security, Shinjuku, Tokyo, Japan; 6 AMR Clinical Reference Center, National Center for Global Health and Medicine, Japan Institute for Health Security, Shinjuku, Tokyo, Japan; 7 Hospital Infection Control Center, Oita University Hospital, Oita, Japan; 8 Department of Clinical Laboratory, Kyoto University Hospital, Kyoto, Japan; 9 Department of Biostatistics and Bioinformatics, Interfaculty Initiative in Information Studies, The University of Tokyo, Tokyo, Japan; 10 Faculty of Medicine and Health Sciences, University of Antwerp, Antwerp, Belgium; 11 Division of Infectious Disease and Hospital Epidemiology, Saga University Hospital, Saga, Japan; 12 Department of Infectious Diseases, Graduate School of Medical Sciences, Nagoya City University, Nagoya, Aichi, Japan; 13 Department of Infectious Diseases, Japanese Red Cross Narita Hospital, Chiba, Japan; 14 Infection Prevention and Control Department, Yokohama City University Hospital, Kanagawa, Japan; 15 Department of Infectious Diseases, Japanese Red Cross Medical Center, Tokyo, Japan; 16 Department of Infectious Diseases, The University of Tokyo Hospital, Tokyo, Japan; 17 Department of Infection Control and Prevention, Osaka University Hospital, Osaka, Japan

**Keywords:** antimicrobial resistance, carbapenemase, carbapenems, genomic cluster, transmission

## Abstract

We aimed to identify genomically defined strain transmission events of carbapenem-resistant Gram-negative bacteria (CRGNB) in hospitals, estimate species-specific transmission rates and identify risk factors for transmission. The study included 708 patients from a Japanese prospective multicentre cohort study, the MultiDrug-Resistant organisms clinical research network, from whom at least one CRGNB isolate was collected between 2019 and 2024 (750 isolates in total). Clinical data were retrieved from electronic medical records, and microbiological analyses were performed at a central reference laboratory. Transmission events were determined via whole-genome sequencing data and single-nucleotide polymorphism-based genomic clustering. We identified 37 genomic clusters associated with intrahospital transmission and 1 associated with interhospital transmission. Ninety-four patients (13%) carried at least one isolate involved in intrahospital transmission, and each transmission event involved two to six patients. Patients with *Acinetobacter* spp. (60% involved in intrahospital transmission), *Enterobacter cloacae* complex (23%) and *Klebsiella oxytoca* complex (59%) were significantly associated with intrahospital transmission and were considered high-risk species groups. Patients with *Enterobacterales* isolates carrying acquired carbapenemase genes had higher intrahospital transmission rates relative to those without (31% vs. 2%). In multivariable analysis, independent risk factors for patients with intrahospital transmission were overlap in ward and time with other CRGNB-positive patients, carriage of high-risk species groups and acquired carbapenemase genes. In this Japanese multicentre cohort, intrahospital transmission of CRGNB was associated with both microbiological characteristics and patient- and healthcare-related factors, underscoring the need for targeted surveillance and infection prevention strategies.

**Impact Statement**

Understanding how carbapenem-resistant Gram-negative bacteria (CRGNB) spread within hospitals is essential for effective infection prevention, yet comparative genomic data across multiple species have been limited. By integrating whole-genome sequencing with phenotypic, epidemiological and clinical data from a multicentre cohort in Japan, this study provides species-specific estimates of intrahospital transmission and identifies key microbiological risk factors, including high-risk species groups and acquired carbapenemase genes. In contrast to previous studies that largely focused on single species or broad categories such as carbapenemase-producing *Enterobacterales*, this study enables a cross-species, comparative assessment of transmission risk across diverse CRGNB. These findings advance our understanding of CRGNB transmission dynamics and support the implementation of risk-stratified infection control strategies to optimize the use of limited infection control resources.

## Data Summary

All genome sequence data analysed in this study are publicly available in the NCBI/DDBJ/EMBL databases under BioProject accession numbers PRJDB37866, PRJDB42112, PRJNA1040422, PRJNA1081165 and PRJNA1452582. Accession numbers for sequencing reads and genome assemblies of all isolates are provided in Dataset 1. Of these, 376 isolates were newly sequenced as part of the present study.

## Introduction

A global increase in infections due to carbapenem-resistant Gram-negative bacteria (CRGNB), including *Enterobacterales*, *Pseudomonas aeruginosa* and *Acinetobacter* spp., has become a critical health threat and a leading cause of morbidity and mortality [1,2]. CRGNB are usually resistant to most other *β*-lactams and frequently exhibit multidrug resistance (MDR), further limiting treatment options [1].

The major mechanism of carbapenem resistance is the production of carbapenemase, driven by clonal expansion of high-risk lineages and, in many species, horizontal spread of acquired carbapenemase genes across species [3,4], together fueling the global rise of carbapenem-resistant *Enterobacterales* (CRE), *P. aeruginosa* and *Acinetobacter baumannii* complex [1,4]. Certain nonfermenting Gram-negative bacteria (GNB), such as *Stenotrophomonas maltophilia*, naturally produce chromosomally encoded carbapenemases, leading to intrinsic resistance that is restricted to the species in which they are encoded [5]. Acquired carbapenemase genes are typically associated with mobile genetic elements such as transposons and integrons and are often carried on plasmids, although they may also be integrated into the chromosome [1].

Nosocomial transmission contributes to the increasing prevalence of CRGNB [6]. Whole-genome sequencing (WGS) analysis with SNP-level resolution now enables the precise identification of transmission events, revealing hidden clusters previously undetected by conventional epidemiological methods [7,8]. Although the transmission risk has been reported to differ by age, contact with colonized individuals, bacterial species and carbapenemase type [9,10], these existing data are limited to single species or carbapenemase-producing *Enterobacterales* (CPE). Consequently, the broader transmission dynamics and drivers of CRGNB, including species-specific differences, have not been systematically evaluated. Understanding microbiological and clinical risk factors across a broader range of CRGNB will be important for refining infection control priorities.

In this study, we characterized the transmission of CRGNB in Japanese hospitals by applying WGS analysis to define genomic transmission events, estimate species-specific transmission rates and identify risk factors associated with transmission.

## Methods

### Study design

This study is a post-hoc analysis of a prospective multicentre CRGNB study conducted by the MultiDrug-Resistant organisms clinical research network. Preliminary findings from an earlier subset of this cohort (2019–2022) were previously reported [11]. The present analysis includes the complete dataset collected between 2019 and 2024. Hospitalized patients from whom CRGNB isolates were cultured from any clinical specimen between April 2019 and March 2024 were enrolled across 11 tertiary care facilities in Japan. For each patient, the first episode in which CRGNB was detected during the index hospitalization was included, regardless of whether it represented infection or colonization. If the initial episode was considered colonization, all episodes were included until an episode represented infection. Transmission events, both within and between healthcare facilities, were identified by WGS analysis. Patients were then classified into two groups: those with transmitted isolates and those without. Only the first episode was included for comparisons of clinical and microbiological characteristics. This study is reported in accordance with the STROBE reporting guidelines [12].

### Patient data

Patient data were extracted from medical records. We examined the epidemiological data to assess whether their hospitalizations overlapped in time and/or ward location at the time of CRGNB detection, as an indicator of potential epidemiological linkage. Detailed clinical, microbiological and genomic methods are described in the Supplementary methods.

### Microbiological and genomic analysis

Eligibility screening was performed by confirming resistance to meropenem or imipenem using disc diffusion testing following CLSI M100-Ed31 [13]. Antimicrobial susceptibility was determined by broth microdilution and interpreted using the CLSI guidelines [13]. MDR, extensive drug resistance (XDR) and difficult-to-treat resistance (DTR) were defined as previously described [14,15].

WGS was performed with NextSeq 1000 or 2000 (Illumina, Inc., San Diego, CA, USA) using 300-cycle paired-end sequencing, as previously described [11]. Draft genomes were obtained using SPAdes version 3.15.5. Genomes were included if they met the following quality control criteria: depth of coverage >30×, N50 >30 kb, <500 contigs and CheckM2 estimates of contamination of <5% and completeness of >90% [16]. Species identification was performed using GTDB-Tk v2.4.1 [17], and the species were grouped into genus or complex levels, considering clinical relevance and species identification reliability in routine laboratory practice (Table S1, available in the online Supplementary Material). Multilocus sequence typing was performed according to public schemes (http://pubmlst.org). For *Acinetobacter* spp., the Pasteur scheme was used. Subspecies-level genomic clustering was performed using PopPUNK [18] for species with available databases (*Acinetobacter* spp., *Escherichia coli*, *Klebsiella pneumoniae* complex, *P. aeruginosa* and *Stenotrophomonas* spp.), avoiding the inaccuracies of sequence type (ST)-based grouping (e.g. single-locus variants may be assigned to different groups). Antimicrobial resistance (AMR) genes were identified using AMRFinderPlus [19]. Insertion sequences (ISs) and transposons were identified by blast searches against the TnCentral [20] and ISFinder databases [21], using thresholds of ≥90% sequence identity and ≥80% query coverage. The number of plasmids was estimated by using MOB-suite [22] based on MOB-cluster assignments, wherein each cluster was considered to represent a distinct plasmid unit. We used an SNP-based whole-genome comparison approach to maximize the resolution and sensitivity of cluster detection. We used ska (https://github.com/simonrharris/SKA) and performed a reference-free, kmer-based calculation of pairwise SNPs using trimmed reads to enable comparisons across diverse species and subspecies-level genomic groups without reliance on a fixed alignment length, unlike conventional core SNP analyses that require a closely related, high-quality reference genome. Genomes were clustered by a single linkage algorithm if they shared >90% split kmers and differed by equal to or fewer than the species-specific SNP thresholds according to the previous reports: *Acinetobacter* spp., 13 [23]; *Citrobacter* spp., 19 [24]; *E. coli*, 15 [24]; *Enterobacter cloacae* complex, 13 [24]; *K. pneumoniae* complex, 12 [24]; *K. oxytoca* complex, 17 [24]; *P. aeruginosa*, 15 [25]. Other *Enterobacterales* species, other nonfermenters and *Aeromonas* spp. were clustered according to the lowest threshold among *Enterobacterales*, nonfermenters and all the species listed above (12, 13 and 12, respectively). The genome data used in this study, including 376 newly sequenced isolates, are summarized in Dataset 1. Transmission rates were calculated as the percentage of patients with at least one CRGNB isolate involved in transmissions defined by genomic clustering.

### Statistical analysis

Continuous variables were analysed with a two-tailed Mann–Whitney U test. Categorical variables were compared using Fisher’s exact test. To analyse risk factors for the transmission of CRGNB, we used a generalized linear mixed-effects logistic regression model, including the following independent variables: time from admission to culture, transfer from another hospital, overlap in ward and time with other CRGNB-positive patients, antimicrobial therapy, non-antimicrobial medical exposure, high-risk species groups, acquired carbapenemase genes and MDR. We used random intercepts to account for clustering by species and facility. We considered *P* values of <0.05 statistically significant. Statistical analyses were performed using R software version 4.2.3 (https://cran.r-project.org).

## Results

A total of 708 patients with 750 CRGNB isolates from whom clinical and WGS data were available were analysed (Fig. S1). These 750 isolates represented 53 species, which were grouped into 17 species groups (Table S1 and Dataset 1).

### Distribution of SNPs

**Fig. 1.**
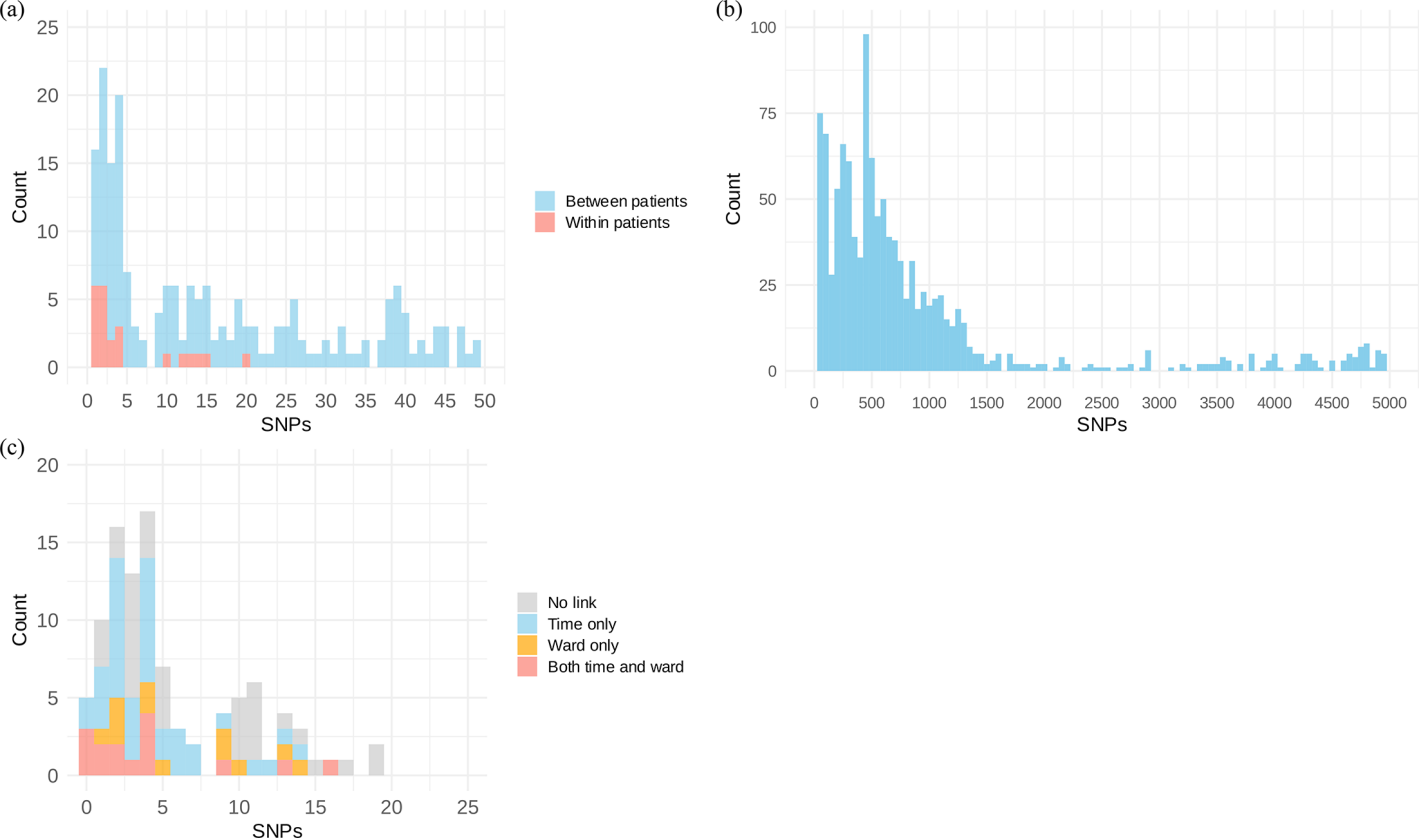
Distribution of pairwise SNP distances among carbapenem-resistant Gram-negative bacteria isolates belonging to the same species or subspecies cluster. Panels (a) and (b) show pairwise SNP distances <50 and <5,000, respectively, among 637 isolates obtained from 600 patients. Among 3,216 pairwise comparisons, 27 were calculated within patients [range, 0–20; median 2; interquartile range (IQR), 1–4 SNPs] and are shown in panel (a). The remaining 3,189 pairwise comparisons between patients ranged from 0 to 41,723 SNPs (median 12,731; IQR, 612–23,721 SNPs). Panel (c) displays the distribution of 104 pairwise SNPs between patients considered to represent intrahospital transmission according to the presence or absence of epidemiological links. A total of 100 isolates obtained from 94 patients that formed 37 genomic clusters within each hospital were included. Alt text: histograms show the number of isolate pairs and the corresponding number of SNPs between them.

PopPUNK analysis divided 549 isolates from 17 species into 187 subspecies clusters. Pairwise SNP distances were calculated for 637 isolates, including 447 isolates belonging to 85 subspecies clusters (each containing more than 1 isolate) and 190 isolates from 25 species that were classified at the species level and were not assigned to subspecies clusters. Twenty patients had multiple isolates of the same species, collected 1–453 days apart, with pairwise SNP distances within patients ranging from 0 to 20, which were significantly lower than the distances between patients (*P*<0.001; Fig. 1a, b). Among these, 14 patients had multiple *P. aeruginosa* isolates collected 1–90 days apart (Fig. S2). In this subset, the pairwise SNP distances ranged from 0 to 20, with the 95th percentile at 15.

### Genomic clustering

Genomic clustering using species-specific thresholds defined 555 clusters. Among 56 genomic clusters with multiple isolates, 18 were restricted to the same patient, 37 were associated with intrahospital transmission, and 1 was associated with interhospital transmission. The relationships of the isolates within the 38 transmission-associated genomic clusters are shown in Fig. S3. Ninety-four patients (13%) carried at least one isolate involved in intrahospital transmission (Table 1). The number of patients involved in intrahospital transmissions ranged from 2 to 6 (median, 2; IQR, 2–3; Table 1). The median of the genomic cluster-level mean durations between admission and isolate collection was 37.5 days (IQR, 17–51.7; Table S2). Four genomic clusters had a mean admission-to-isolate collection duration of ≤2 days; however, all patients associated with these clusters had at least one medical exposure before admission. *P. aeruginosa* and *E. cloacae* complex were the most frequent species groups associated with intrahospital transmission (Table 1). Among strain pairs considered to represent intrahospital transmission, overlaps in both time and ward were identified in 14%, temporal overlap within the same hospital but different wards in 12% and ward overlap without temporal overlap in 38%, whereas no overlap occurred in either time or ward in 37% (Fig. 1c). Two *P. aeruginosa* isolates separated by 15 SNPs from 2 patients were considered to represent interhospital transmission. However, these patients were hospitalized in distant regions and had no epidemiological linkage, and the time between admission and isolate collection was 77 days for both patients (Table S2).

**Table 1.** Number of patients with intrahospital transmission of carbapenem-resistant Gram-negative bacteria by species group

Species group	No. of patients	No. of isolates	Patients with intra hospital transmission (transmission rate)	No. of intrahospital genomic clusters according to cluster size (no. of patients)					
				Any	2	3	4	5	6
*Enterobacterales*									
*Citrobacter* spp.	17*	18	2 (12%)	1	1	0	0	0	0
*E. cloacae* complex	60*	62	14^†^ (23%)	6	4	2	0	0	0
*E. coli*	22*	22	0 (0%)	0	0	0	0	0	0
*Klebsiella aerogenes*	23	24	0 (0%)	0	0	0	0	0	0
*Klebsiella oxytoca* complex	17*	17	10 (59%)	3	1	1	0	1	0
*K. pneumoniae* complex	44*	46	7 (16%)	3	2	1	0	0	0
*Proteus mirabilis*	3	3	0 (0%)	0	0	0	0	0	0
*Serratia* spp.	5	5	2 (40%)	1	1	0	0	0	0
Nonfermenters									
*Acinetobacter* spp.	10	10	6 (60%)	1	0	0	0	0	1
*Chryseobacterium* spp.	18	18	0 (0%)	0	0	0	0	0	0
*Elizabethkingia* spp.	8*	8	0 (0%)	0	0	0	0	0	0
*Metapseudomonas otitidis*	6	6	0 (0%)	0	0	0	0	0	0
*P. aeruginosa*	360	391	52† (14%)	21	16	2	1	2	0
*Stenotrophomonas* spp.	79*	80	2 (3%)	1	1	0	0	0	0
*Aeromonas* spp.	40	40	0 (0%)	0	0	0	0	0	0
Total	708	750	94 (13%)	37	26	6	1	3	1

*Four patients each had two isolates of different species on the same day: *Citrobacter* spp. and *K. oxytoca* complex; *E. cloacae* complex and *K. oxytoca* complex; *E. coli* and *K. pneumoniae* complex; *Elizabethkingia* spp. and *Stenotrophomonas* spp.

†One patient was involved in intrahospital transmissions of both *Enterobacter asburiae* (cluster size, *n*=3) and *P. aeruginosa* (cluster size, *n*=2).

**Fig. 2.**
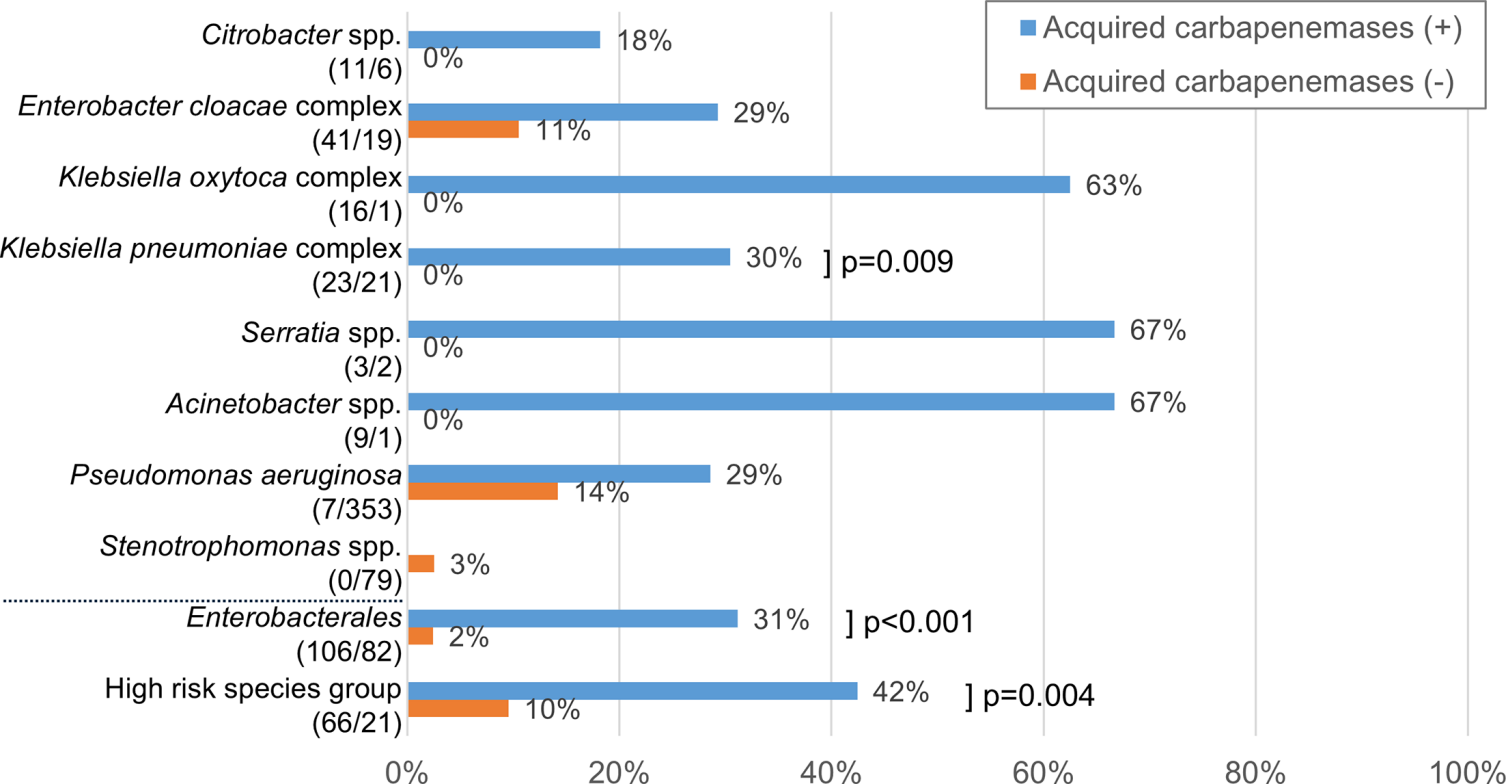
Intrahospital transmission rates of patients with carbapenem-resistant Gram-negative bacteria, on the basis of the presence or absence of acquired carbapenemase genes. The numbers in parentheses under the species group name represent the number of patients with isolates carrying acquired carbapenemase genes versus those without. The high-risk species group included *Acinetobacter* spp., *E. cloacae* complex and *K. oxytoca* complex. Species groups with no transmission events are not shown in this figure. Alt text: bar charts show higher transmission rates in patients with isolates carrying acquired carbapenemase genes compared with those without, stratified by species or species group.

The STs and carbapenemase genes of the isolates belonging to the 38 genomic clusters are detailed in Table S2. Multiple genomic clusters were found in *K. oxytoca* complex ST43 (*n*=2), *E. cloacae* complex ST133 (*n*=2) and *P. aeruginosa* ST164 (*n*=3), ST313 (*n*=3) and ST186 (*n*=2). Among the 14 *Enterobacterales* genomic clusters, 13 (93%) were associated with acquired carbapenemase genes (*bla*_IMP-1_, *n*=9; *bla*_GES-5_, *n*=2; *bla*_IMP-60_, *n*=1; *bla*_GES-24_, *n*=1); conversely, only 1 of 22 *P*. *aeruginosa* genomic clusters (5%) carried *bla*_IMP-7_. The only *Acinetobacter* spp. genomic cluster was associated with *bla*_IMP-1_. Intrahospital transmission rates were higher in patients with isolates carrying acquired carbapenemase genes than in those without such genes across all species groups except *S. maltophilia* (Fig. 2). This difference was only statistically significant for *K. pneumoniae* complex (30% vs. 0%), *Enterobacterales* (31% vs. 2%) and high-risk species groups (42% vs. 10%; defined below).

Exploratory isolate-level genomic comparative analyses were performed to characterize isolates involved in intrahospital transmission. Among all CRGNB isolates, transmitted isolates harboured higher numbers of AMR genes (including those targeting aminoglycosides, *β*-lactams, phenicols, quaternary ammonium compounds and sulphonamide/trimethoprim), as well as higher numbers of plasmids and ISs, and were more likely to carry the class I integrase genes compared with non-transmitted isolates (Table S3). Species group-specific analyses were additionally performed for *Enterobacterales* and *P. aeruginosa*. In *Enterobacterales*, compared with non-transmitted isolates, transmitted isolates harboured higher numbers of AMR genes that target quaternary ammonium compounds and were more likely to carry the class 1 integrase genes. In *P. aeruginosa*, no significant differences were observed between transmitted and non-transmitted isolates.

### Risk factors for patients with intrahospital transmission

Table 2 summarizes the clinical characteristics of patients with and without intrahospital transmission. In univariable analyses of patient backgrounds, significant risk factors for patients with intrahospital transmission included time from admission to culture >2 days (OR, 2.2; 95% CI, 1.2–4.0), transfer from another hospital (OR, 1.8; CI, 1.1–3.0), overlap in ward and time with other CRGNB-positive patients (OR, 1.9; CI, 1.2–3.0), overlap in ward and time with patients with the same species (OR, 2.3; CI, 1.5–3.7), antimicrobial therapy within 14 days (OR, 2.2; CI, 1.2–4.3), intravenous therapy within 30 days (OR, 2.3; CI, 1.4–4.1) and surgery within 30 days (OR, 1.7; CI, 1.1–2.7). We explored alternative definitions of ward/time overlap (Table S4). Time overlap alone and ward or time overlap classified nearly all patients, regardless of transmission status, as positive. Ward overlap alone and overlap in ward and time were both significantly more frequent in transmitted than in non-transmitted patients, with minimal differences in their proportions between the two definitions. Similar patterns were observed in analyses restricted to the same species. Univariable analyses of outcomes revealed that patients with intrahospital transmission had a longer hospital stay (median 84.5 vs. 50.5 days; *P*<0.001). Discharge type differed between the groups; specifically, discharge to home was less frequently observed, whereas transfer to another hospital was more frequently observed, in patients with intrahospital transmission. In terms of microbiological characteristics, significant risk factors for intrahospital transmission included skin and soft tissue specimens (OR, 3.5; CI, 1.8–6.9), acquired carbapenemase genes (OR, 4.9; CI, 3.0–7.9) and MDR (OR, 6.0; CI, 2.2–18.3) (Table 3). Conversely, the presence of naturally occurring carbapenemase genes without acquired genes was significantly associated with patients who did not experience intrahospital transmission. *Enterobacterales* as a whole, as well as *Acinetobacter* spp., were significantly associated with intrahospital transmission. Within *Enterobacterales*, *E. cloacae* complex and *K. oxytoca* complex were significantly associated with intrahospital transmission; these, together with *Acinetobacter* spp., were defined as the high-risk species groups (OR, 4.7; CI, 2.8–8.1). In contrast, *Aeromonas* spp. and *Stenotrophomonas* spp. were significantly associated with patients who did not experience intrahospital transmission.

**Table 2.** Comparison of clinical characteristics in patients with and without intrahospital transmission of carbapenem-resistant Gram-negative bacteria

	N (%)			
Characteristic	Transmitted (*n*=94)	Non-transmitted (*n*=614)	Odds ratio (95% CI)	*P* value
Age [median (IQR)], year	69 (57.25–77.75)	71 (58–78)		0.287
Male sex	64 (68%)	377 (61%)	1.3 (0.8–2.2)	0.253
Time from admission to culture, days	29.5 (9–59.25)	18 (2–43)		0.008
Time from admission to culture >2 days	80 (85%)	446 (73%)	2.2 (1.2–4.0)	0.011
Preadmission origin				
Home	68 (72%)	475 (77%)	0.8 (0.5–1.3)	0.295
Transfer from another hospital	25 (27%)	104 (17%)	1.8 (1.1–3.0)	0.031
Long-term care	1 (1%)	32 (5%)	0.2 (0.0–1.1)	0.109
Birth at hospital	0	3 (0.5%)	0 (0–11.2)	1
Overseas travel history within 3 months	0	11 (3%)	0 (0–2.1)	0.384
Location at the time of culture collection				
Ward	72 (77%)	470 (77%)	1.0 (0.6–1.7)	1
ICU	18 (19%)	94 (15%)	1.3 (0.7–2.3)	0.362
Outpatient	4 (4%)	46 (7%)	0.5 (0.2–1.6)	0.385
Overlap in ward and time				
With other CRGNB-positive patients	59 (63%)	291 (47%)	1.9 (1.2–3.0)	0.006
With patients with the same species	42 (45%)	158 (26%)	2.3 (1.5–3.7)	<0.001
Comorbidities				
Myocardial infarction	6 (6%)	40 (7%)	1.0 (0.4–2.3)	1
Congestive heart failure	4 (4%)	56 (9%)	0.4 (0.1–1.2)	0.161
Peripheral vascular disease	4 (4%)	22 (4%)	1.2 (0.4–3.7)	0.767
Cerebrovascular disease	17 (18%)	83 (14%)	1.4 (0.8–2.5)	0.265
Dementia	6 (6%)	37 (6%)	1.1 (0.4–2.6)	0.819
Chronic obstructive pulmonary disease	7 (7%)	52 (8%)	0.9 (0.4–2.0)	0.843
Connective tissue disease	11 (12%)	55 (9%)	1.3 (0.7–2.7)	0.444
Peptic ulcer disease	1 (1%)	8 (1%)	0.8 (0.0–5.3)	1
Any liver disease	9 (10%)	44 (7%)	1.4 (0.6–2.9)	0.401
Diabetes mellitus	25 (27%)	148 (24%)	1.1 (0.7–1.9)	0.607
Hemiplegia	5 (5%)	18 (3%)	1.9 (0.6–5.0)	0.214
Any renal diseases	9 (10%)	50 (8%)	1.2 (0.6–2.6)	0.688
Localized solid tumour	15 (16%)	140 (23%)	0.6 (0.4–1.2)	0.143
Metastatic solid tumour	9 (10%)	65 (11%)	0.9 (0.4–1.9)	0.858
Leukaemia	2 (2%)	30 (5%)	0.4 (0.1–1.7)	0.295
Lymphoma	3 (3%)	21 (3%)	0.9 (0.2–3.1)	1
Charlson Comorbidity Index [median (IQR)]	2 (1–4)	2 (1–4)		0.523
Medical exposure				
Antimicrobial therapy within 14 days	82 (87%)	464 (76%)	2.2 (1.2–4.3)	0.012
Non-antimicrobial medical exposure	89 (95%)	529 (86%)	2.9 (1.1–7.6)	0.019
Prior admission within 30 days	41 (44%)	214 (35%)	1.4 (0.9–2.3)	0.107
Haemodialysis within 30 days	3 (3%)	41 (7%)	0.5 (0.1–1.5)	0.253
ICU stay within 30 days	27 (29%)	171 (28%)	1.0 (0.6–1.7)	0.902
Intravenous therapy within 30 days	76 (81%)	396 (64%)	2.3 (1.4–4.1)	0.001
Endoscopy within 30 days	16 (17%)	110 (18%)	0.9 (0.5–1.7)	1
Surgery within 30 days	34 (36%)	155 (25%)	1.7 (1.1–2.7)	0.033
Mechanical ventilation	27 (29%)	120 (20%)	1.7 (1.0–2.7)	0.055
Immunosuppressive state	32 (34%)	239 (39%)	0.8 (0.5–1.3)	0.425
Glucocorticoids	27 (29%)	186 (30%)	0.9 (0.6–1.5)	0.810
Antitumour chemotherapy	7 (7%)	70 (11%)	0.6 (0.3–1.4)	0.290
Biological agents	3 (3%)	15 (2%)	1.3 (0.3–4.4)	0.721
Immunosuppressants	10 (11%)	72 (12%)	0.9 (0.4–1.8)	0.864
Solid organ transplantation	7 (7%)	47 (8%)	1.0 (0.4–2.2)	1
Haematopoietic stem cell transplantation	1 (1%)	18 (3%)	0.4 (0.0–2.3)	0.494
Human immunodeficiency virus infection	0	1 (0.2%)	0 (0–124.1)	1
Disease status				
Infection	48 (51%)	264 (43%)	1.4 (0.9–2.2)	0.148
Type of infection*				
Catheter-related infection	5 (10%)	18 (7%)	1.6 (0.5–4.5)	0.371
Respiratory tract infection	11 (23%)	80 (30%)	0.7 (0.3–1.4)	0.388
Urinary tract infection	7 (15%)	48 (18%)	0.8 (0.3–1.8)	0.682
Intra-abdominal infection	7 (15%)	40 (15%)	1.0 (0.4–2.3)	1
Surgical site infection	8 (17%)	20 (8%)	2.4 (1.0–6.1)	0.054
Bloodstream infection of unknown source	3 (6%)	18 (7%)	0.9 (0.2–3.1)	1
Others	7 (15%)	40 (15%)	1.0 (0.4–2.3)	1
Outcomes				
Length of hospital stay [median (IQR)], days	84.5 (36.5–138.25)	50.5 (24–95.25)		<0.001
30-day mortality†	15 (16%)	85 (14%)	1.2 (0.6–2.1)	0.635
Readmission within 90 days‡	19 (20%)	149 (24%)	0.8 (0.4–1.3)	0.436
Disposition				
Home‡	30 (32%)	304 (50%)	0.5 (0.3–0.8)	0.001
Transfer to another hospital‡	36 (38%)	155 (25%)	1.8 (1.1–2.9)	0.012
Long-term care‡	4 (4%)	22 (4%)	1.2 (0.4–3.6)	0.767
Death‡	24 (26%)	131 (21%)	1.3 (0.8–2.1)	0.353

* Comparison restricted to patients with infection (transmitted, *n*=48; non-transmitted, *n*=264). Infection types were classified into mutually exclusive categories.

† Comparison restricted to patients with available mortality data (transmitted, *n*=93; non-transmitted, *n*=598).

‡ Comparison restricted to patients who had been discharged (transmitted, *n*=94; non-transmitted, *n*=612).

CRGNB, carbapenem-resistant Gram-negative bacteria; ICU, intensive care unit; IQR, interquartile range.

**Table 3.** Comparison of microbiological characteristics in carbapenem-resistant Gram-negative bacteria isolates from patients with and without intrahospital transmission

	N (%)			
Characteristic	Transmitted* (*n*=94)	Non-transmitted* (*n*=614)	Odds ratio (95% CI)	*P* value
Culture source				
Lower respiratory tract	36 (38%)	241 (39%)	1.0 (0.6–1.5)	0.910
Urine	13 (14%)	109 (18%)	0.7 (0.4–1.4)	0.383
Blood	14 (15%)	78 (13%)	1.2 (0.6–2.3)	0.514
Bile	4 (4%)	60 (10%)	0.4 (0.1–1.1)	0.119
Skin and soft tissue	14 (15%)	29 (5%)	3.5 (1.8–6.9)	<0.001
Stool	3 (3%)	40 (7%)	0.5 (0.1–1.5)	0.253
Intra-abdominal	2 (2%)	21 (3%)	0.6 (0.1–2.6)	0.756
Others	8 (9%)	36 (6%)	1.5 (0.6–3.4)	0.356
Species group				
*Enterobacterales*	35 (37%)	153 (25%)	1.8 (1.1–2.9)	0.017
*Citrobacter* spp.	2 (2%)	15 (2%)	0.9 (0.1–3.7)	1
*E. cloacae* complex	14 (15%)	45 (7%)	2.2 (1.1–4.3)	0.025
*E. coli*	0	21 (3%)	0 (0–1.2)	0.096
*K. aerogenes*	0	23 (4%)	0 (0–1.1)	0.059
*K. oxytoca* complex	10 (11%)	6 (1%)	12.0 (4.0–34.1)	<0.001
*K. pneumoniae* complex	7 (7%)	37 (6%)	1.3 (0.5–3.0)	0.645
*P. mirabilis*	0	3 (0.5%)	0 (0–11.2)	1
Nonfermenters	59 (63%)	421 (69%)	0.8 (0.5–1.2)	0.286
*Acinetobacter* spp.	6 (6%)	4 (1%)	10.3 (2.7–39.8)	<0.001
*Chryseobacterium* spp.	0	18 (3%)	0 (0–1.5)	0.152
*Elizabethkingia* spp.	0	7 (1%)	0 (0–3.9)	0.603
*M. otitidis*	0	6 (1%)	0 (0–4.6)	1
*P. aeruginosa*	51 (54%)	309 (50%)	1.2 (0.8–1.8)	0.507
*Serratia* spp.	2 (2%)	3 (0.5%)	4.4 (0.5–28.7)	0.133
*Stenotrophomonas* spp.	2 (2%)	77 (13%)	0.2 (0.0–0.6)	0.001
*Aeromonas* spp.	0	40 (7%)	0 (0–0.6)	0.006
High-risk species groups†	30 (32%)	55 (9%)	4.7 (2.8–8.1)	<0.001
AMR				
Carbapenemase genes, acquired	40 (43%)	81 (13%)	4.9 (3.0–7.9)	<0.001
*bla*_IMP_‡	33 (35%)	67 (11%)	4.4 (2.7–7.2)	<0.001
*bla*_GES_§	7 (7%)	1 (0.2%)	48.9 (6.7–1096.9)	<0.001
*bla*_NDM_¶	0	10 (2%)	0 (0–2.7)	0.374
Other carbapenemase genes#	0	3 (0.5%)	0 (0–11.2)	1
Carbapenemase genes, naturally occurring only	2 (2%)	113 (18%)	0.1 (0.0–0.4)	<0.001
MDR	90 (96%)	484 (79%)	6.0 (2.2–18.3)	<0.001
XDR	45 (48%)	273 (44%)	1.1 (0.7–1.8)	0.578
DTR	22 (23%)	176 (29%)	0.8 (0.4–1.3)	0.325

*Only the first isolate per patient was included; in patients with transmission, the first isolate within the transmission cluster was selected. One patient experienced intrahospital transmission of *E. asburiae* and *P. aeruginosa*; only *E. asburiae* (the first event) is included in this table.

†*Acinetobacter* spp., *E. cloacae* complex and *K. oxytoca* complex were included.

‡Patients with transmission had isolates carrying *bla*_IMP-1_ (*n*=28), *bla*_IMP-60_ (*n*=3) and *bla*_IMP-7_ (*n*=2), whereas those without transmission had isolates carrying *bla*_IMP-1_ (*n*=59), *bla*_IMP-11_ (*n*=5), *bla*_IMP-6_ (*n*=2) and *bla*_IMP-7_ (*n*=1).

§Patients with transmission had isolates carrying *bla*_GES-5_ (*n*=5) and *bla*_GES-24_ (*n*=2), whereas those without transmission had isolates carrying *bla*_GES-5_ (*n*=1).

¶Patients without transmission had isolates carrying *bla*_NDM-5_ (*n*=6), *bla*_NDM-1_ (*n*=3) and *bla*_NDM-7_ (*n*=1).

#Patients without transmission had isolates carrying *bla*_KPC-2_, *bla*_OXA-48_ and *bla*_TMB-1_ (*n*=1 each).

DTR, difficult-to-treat resistance; MDR, multidrug resistance; XDR, extensive drug resistance.

Multivariable analysis indicated that overlap in ward and time with other CRGNB-positive patients, high-risk species groups and acquired carbapenemase genes were significant risk factors for intrahospital transmission (Table 4).

**Table 4.** Multivariable analysis of risk factors for intrahospital transmission of carbapenem-resistant Gram-negative bacteria*

Variables	Odds ratio (95% CI)	*P* value
Time from admission to culture >2 days	1.1 (0.5–2.3)	0.770
Transfer from another hospital	1.6 (0.9–2.9)	0.104
Overlap in ward and time with other CRGNB-positive patients	2.2 (1.3–3.8)	0.002
Antimicrobial therapy within 14 days	2.0 (1.0–4.3)	0.061
Non-antimicrobial medical exposure	2.0 (0.7–5.8)	0.186
High-risk species groups	6.0 (1.4–26.6)	0.018
Acquired carbapenemases	4.9 (2.0–11.9)	<0.001
MDR	2.2 (0.6–8.2)	0.239

*A generalized linear mixed-effects model was used, including species group and facility as random effects. Random intercept variances for species group and facility were 0.65 (sd, 0.81) and 0.06 (sd, 0.25), corresponding to intraclass correlation coefficients of 0.17 and 0.02, respectively.

CRGNB, carbapenem-resistant Gram-negative bacteria; MDR, multidrug resistance.

## Discussion

This genomic transmission analysis of patients with CRGNB revealed that intrahospital transmission events occurred at different rates across species and highlighted specific species and the presence of acquired carbapenemase genes as microbiological risk factors. In a previous hospital-wide investigation based on 2,752 isolates identified by an electronic outbreak detection system, a genomic analysis that included all clinical isolates irrespective of species or AMR profiles identified nosocomial transmission in 11% of patients [8]. Studies focusing on CRGNB have reported transmission rates of 8–18% for CRE [26,27], 42–60% for CPE [9,10] and 38% for carbapenem-resistant *A. baumannii* [23]. Although these studies differ in design, definitions of transmission and analytic approaches, the transmission rates observed in our study (19%, 31% and 60%, respectively; Table 1 and Fig. 2) do not markedly differ from those reported in previous studies. Species-specific transmission rates within *Enterobacterales* or for other species have not yet been reported. Genomic clusters of CRE and CPE have been reported to have medians of 2–3 patients, with relatively large outbreak clusters with 5–20 patients occasionally observed [9,10, 26,28]. Consistent with these findings, most clusters in our study included two patients.

A regional genomic transmission study of CPE reported that transmission risk was significantly elevated even without temporal or ward overlap if patients were admitted to the same hospital; conversely, clinical discipline and medical procedures were not significant risk factors [10]. In our study, the available epidemiological data were limited to hospital location at specimen collection and length of stay; shared exposures such as rehabilitation or endoscopic procedures could not be evaluated. Nevertheless, two-thirds of the clustered isolates showed temporal and/or ward overlap (Fig. 1c). Other studies, including those involving non-AMR organisms or Gram-positive cocci, have reported epidemiological links for 26–69% of genomic clusters [7,8, 29,31], similarly indicating the coexistence of hidden intrahospital transmission and community or interhospital acquisition. In this study, clusters detected within 2 days of admission involved patients with prior healthcare exposures, suggesting that recent healthcare exposure may have contributed to acquisition.

Univariable analyses revealed that several clinical factors were significantly associated with intrahospital transmission in patients who already carried CRGNB (Table 2). These factors are also well-known risk factors for acquisition [32], consistent with the notion that transmission is a major mode of acquisition. In this context, acquisition refers to the detection of a resistant organism in a previously negative patient, irrespective of strain relatedness, whereas transmission is defined by genetic relatedness between isolates. In addition to microbiological factors, overlap in ward and time with other CRGNB-positive patients remained significantly associated with transmission in the multivariable model (Table 4), suggesting that both microbiological characteristics and ward-level factors may contribute to transmission. This variable may be interpreted as a proxy indicator of shared ward-level risk, as such overlap was frequently observed in both transmitted and non-transmitted patients. It likely reflects a composite of patient- and healthcare-related factors, including underlying patient characteristics (e.g. chronic conditions and medical exposures) and infection control practices within specific wards. The multivariable model should be interpreted with caution, as the high-risk species group variable was defined in a post-hoc manner and was based on observed transmission patterns; thus, this analysis should be considered exploratory. In addition, the model may reflect the absence of direct measures of infection control practices (e.g. hand hygiene compliance and adherence to contact precautions) or clinical factors directly linked to transmission (e.g. nursing care intensity and number of healthcare staff contacts).

We observed frequent transmission in *Enterobacterales*, when they carried acquired carbapenemase genes, whereas species with intrinsic carbapenemase genes rarely showed transmission. The higher transmissibility of CPE than that of non-carbapenemase-producing CRE aligns with the current epidemiology, where CPE predominate among CRE [33]. In a pan-European analysis of *K. pneumoniae*, approximately half of the CPE isolates were genomically closest to another isolate from the same hospital – a higher frequency than that for non-carbapenemase-producing CRE [34]. To our knowledge, no studies have directly compared transmission rates between patients carrying CRE with and without carbapenemase genes. A prior study reported that CPE acquisition was associated with CRE colonization pressure, whereas non-carbapenemase-producing CRE acquisition was correlated with the duration of carbapenem treatment [35], suggesting that CPE is more often acquired via transmission.

The *E. cloacae* complex, *K. oxytoca* complex and *Acinetobacter* spp. exhibited significant transmission risk in our cohort (Table 3). Similarly, a regional study in Singapore revealed *K. pneumoniae* and *Enterobacter* spp. as high-risk species for transmission among CPE [10]. For several species groups, including *Acinetobacter* spp., the numbers of isolates were limited, and transmission events were sometimes represented by a single cluster (Table S1). Therefore, the observed transmission rates for these species should be interpreted cautiously, as they may reflect isolated outbreaks rather than generalizable species-level characteristics. STs were diverse in our genomic clusters (Table S2), and no dominant clones were identified, reflecting the current epidemiology in Japan, where the prevalence of CRE remains low (~1%) and carbapenemase producers are not yet endemic [36]. Similarly, studies from Singapore, Ireland and the UK reported transmission events across multiple nonmajor STs [24,28]. In contrast, in the USA, where KPC-producing *K. pneumoniae* clonal group 258 predominated among CPEs [37], transmission rates reached 60%, with dissemination both within and between hospitals [9]. High (38–76%) proportions of interhospital transmission have also been reported [9,10, 26, 27], emphasizing the need for infection prevention measures at both the facility and regional levels. In our cohort, however, interhospital transmission was rarely observed, likely because the participating hospitals were geographically distant and patient sharing was minimal.

Given the absence of a standardized approach for defining genomic transmission clusters [3], we applied previously validated methods and species-specific thresholds. Among *P. aeruginosa* isolates, 95% of within-patient pairs differed by fewer than 15 SNPs, which was identical to the thresholds adopted, confirming the validity and compatibility of our approach at least for this species. Single-linkage clustering may link distantly related isolates into the same cluster [38]. Among the 38 genomic clusters identified, 11 involved 3 or more isolates, and only 1 of them contained any isolate pairs that exceeded the pairwise SNP threshold (Fig. S3). This indicates that most clusters were tightly defined and that false-positive detection was minimal.

Genomic comparison between transmitted and non-transmitted isolates (Table S3) should be interpreted with caution, as associations between AMR gene or mobile genetic elements and transmission may partly reflect species- or lineage-specific genomic backgrounds rather than independent effects of individual elements. In fact, most of these associations were not consistently observed in species group-specific analyses, with the exception of AMR genes associated with resistance to quaternary ammonium compounds. Species-specific analyses were limited to *Enterobacterales* and *P. aeruginosa*, as only these groups possessed sufficient numbers of isolates (≥20) for meaningful comparison. The observation that transmitted isolates harboured greater numbers of AMR genes is consistent with the higher prevalence of MDR phenotypes observed among isolates from patients involved in transmission, suggesting the importance of appropriate antimicrobial stewardship across multiple antibiotic classes. In addition, the association between resistance to quaternary ammonium compounds, which are commonly used disinfectants in hospital settings, and transmitted isolates may reflect conditions that facilitate persistence and spread in the healthcare environment, highlighting the importance of environmental control measures in addition to antimicrobial stewardship [39].

This study has several limitations. First, as a post-hoc analysis without active surveillance or environmental sampling, the transmission rate was likely underestimated, and epidemiological links could not be fully assessed. In addition, not all infection and colonization episodes, along with their corresponding isolates, may have been captured, as the original cohort was not designed for all CRGNB events. Furthermore, although consecutive CRGNB cases were intended to be enrolled at each facility, the total number of eligible cases was not systematically recorded across all centres, and the overall enrolment rate could not be determined. Risk factor analysis was also constrained by the lack of detailed patient characteristics and infection control indicators. Second, the sample size was insufficient for robust analyses of several minor species groups. Nevertheless, a major strength of this study is its inclusion of all GNB species, which has not been performed previously. Third, although mobile genetic elements can contribute to the nosocomial spread of carbapenemase genes [10], their transmission was not assessed. Finally, the generalizability of our risk factor analyses may be limited because microbiological features and healthcare settings vary across regions.

In conclusion, we identified intrahospital transmission events of CRGNB at the genomic level in acute-care hospitals in Japan. The transmission rates varied by species and by the presence of acquired carbapenemase genes, both of which were significant risk factors for intrahospital transmission. In addition, overlap in ward and time with other CRGNB-positive patients was also associated with transmission. These findings support the implementation of risk-stratified infection control strategies, including the rapid detection of carbapenemases, to optimize the use of limited infection control resources. Furthermore, real-time genomic surveillance may strengthen these efforts. Continued efforts to reduce intrahospital CRGNB transmission events remain essential.

## Supplementary material

10.1099/mgen.0.001770 Supplementary Material 1.

## Abbreviations

AMR: antimicrobial resistance
CPE: carbapenemase-producing *Enterobacterales*
CRE: carbapenem-resistant *Enterobacterales*
CRGNB: carbapenem-resistant Gram-negative bacteria
DTR: difficult-to-treat resistance
GNB: Gram-negative bacteria
IQR: interquartile range
ISs: insertion sequences
MDR: multidrug resistance
ST: sequence type
WGS: whole-genome sequencing
XDR: extensive drug resistance
