# Supplementary material for: Genomic analysis of intrahospital transmission of carbapenem-resistant Gram-negative bacteria: a multicentre study in Japan
Source: Microb Genom. 2026 Jul 7;12(7):001770. doi: 10.1099/mgen.0.001770 (PMC13340626; doi:10.1099/mgen.0.001770)
Supplement: Supplementary Material 1. [file mgen-12-01770-s001.pdf]

Supplementary materials

Supplementary methods

Study design

For comparisons of clinical and microbiological characteristics, only the first episode was included; if when more than one isolate was detected on the same day, the isolate with the lowest identification number was analysed. This post hoc analysis relied on data collected in a multicentre observational cohort. Selection bias may exist because data collection was not originally designed for the present research question. A sample size calculation was not conducted, as the study size was determined by the number of participants available in the original cohort. The number of missing data points was reported for each variable, and analyses were performed using complete-case analysis. Missing values were not imputed. Sensitivity analyses were not undertaken, as all available clinical and genomic data were included and no alternative analytical assumptions were specified.

Patient data and definitions

Patient data were extracted from medical records and entered into a REDCap database. Patients were followed from the time of hospital admission until discharge or death. The following characteristics were obtained from the database: demographics, preadmission origin, patient location at the time of culture, comorbidities, medical exposure, immunosuppression status, disease status (infection or colonization), clinical focus of infection, culture source, outcomes (including 30-day mortality, length of hospital stay, and discharge disposition), and microbiological data (specimen type and species name).

A patient with a culture positive for CRGNB was considered to have an infection if the specimen was isolated from blood or other normally sterile sources. We used the Centers for Disease Control and Prevention definitions for central line-associated bloodstream infection, respiratory tract infection, urinary tract infection, and surgical site infection (1-4). Patients with cultures from sites other than those listed above were only considered infected if they had evidence of systemic inflammation on the day of the positive culture. Systemic inflammation was defined as an elevated or low white blood cell count (>10,000 or <4,000 cells/μl, respectively) and/or an abnormal body temperature (>37.5°C or <35.5°C) (5). Infection types were classified into mutually exclusive categories based on CDC definitions. Bloodstream infections that were not categorized as central

line-associated bloodstream infection or secondary bloodstream infection attributable to a defined infection focus were categorized as bloodstream infection of unknown source. Among the remaining cases, bloodstream infection cases without an identifiable source were classified into "bloodstream infection of unknown source." Cases not meeting these definitions were categorized as "other."

Isolates were considered to represent colonization if patients did not meet any of the above criteria.

Microbiological analysis

We used a centralized approach for the microbiological analysis (6). All microbiological analyses were performed at the Fujita Health University School of Medicine. Eligibility screening was performed by confirming resistance to meropenem or imipenem using disk diffusion testing following CLSI M100-Ed31 (7). Antimicrobial susceptibility testing was evaluated by broth microdilution using a customized panel (Eiken Chemical, Tokyo, Japan) according to the manufacturer's instructions and was interpreted using the CLSI guidelines (7). With respect to *Enterobacterales* isolates, a tigecycline MIC >0.5 mg/L was considered non-susceptible according to the European Committee on Antimicrobial Susceptibility Testing criteria. MDR, XDR, and difficult-to-treat resistance (DTR) were defined according to previous definitions (8, 9). *Stenotrophomonas maltophilia* was considered DTR (10).

References

1. Centers for Disease Control and Prevention. Bloodstream Infection Event (Central Line-Associated Bloodstream Infection and Non-central Line Associated Bloodstream Infection) 2019 [updated July 16, 2019. Available from: https://www.cdc.gov/nhsn/pdfs/pscmanual/4psc_clabscurrent.pdf.

2. Centers for Disease Control and Prevention. Pneumonia (Ventilator-associated [VAP] and non-ventilator-associated Pneumonia [PNEU]) Event 2019 [updated July 16, 2019. Available from: https://www.cdc.gov/nhsn/pdfs/pscmanual/6pscvapcurrent.pdf.

3. Centers for Disease Control and Prevention. Urinary Tract Infection (Catheter-Associated Urinary Tract Infection [CAUTI] and Non-Catheter-Associated Urinary Tract Infection [UTI]) and Other Urinary 2019 [updated July 16, 2019. Available from: https://www.cdc.gov/nhsn/PDFs/pscManual/7pscCAUTIcurrent.pdf.

4. Centers for Disease Control and Prevention. Surgical Site Infection (SSI) Event 2019 [Available from: https://www.cdc.gov/nhsn/pdfs/pscmanual/9pscssicurrent.pdf.

5. van Duin D, Perez F, Rudin SD, Cober E, Hanrahan J, Ziegler J, et al. Surveillance of carbapenem-resistant *Klebsiella pneumoniae*: tracking molecular epidemiology and outcomes through a regional network. Antimicrob Agents Chemother. 2014;58(7):4035-41. DOI:10.1128/AAC.02636-14

6. Saito S, Sakurai A, Matsumura Y, Uemura K, Hase R, Kato H, et al. Molecular epidemiology and patient outcome of carbapenem-resistant Enterobacterales, *Pseudomonas aeruginosa* and *Acinetobacter baumannii* in Japan: a multicenter study from MultiDrug-Resistant organisms clinical research network. JAC Antimicrob Resist. 2025;7(2):dlaf027. DOI:10.1093/jacamr/dlaf027

7. CLSI. Performance Standards for Antimicrobial Susceptibility Testing. 31st ed. CLSI supplement M100. Wayne, PA: Clinical and Laboratory Standards Institute. 2021.

8. Magiorakos AP, Srinivasan A, Carey RB, Carmeli Y, Falagas ME, Giske CG, et al. Multidrug-resistant, extensively drug-resistant and pandrug-resistant bacteria: an international expert proposal for interim standard definitions for acquired resistance. Clin Microbiol Infect. 2012;18(3):268-81. DOI:10.1111/j.1469-0691.2011.03570.x

9. Kadri SS, Adjemian J, Lai YL, Spaulding AB, Ricotta E, Prevots DR, et al. Difficult-to-Treat Resistance in Gram-negative Bacteremia at 173 US Hospitals: Retrospective Cohort Analysis of Prevalence, Predictors, and Outcome of Resistance to All First-line Agents. Clin Infect Dis. 2018;67(12):1803-14. DOI:10.1093/cid/ciy378

10. Echols RM, Tillotson GS. Difficult to Treat: Do We Need a New Definition? Clin Infect Dis. 2019;69(9):1641-2. DOI:10.1093/cid/ciz184

Table S1. Number of patients with intrahospital transmission of carbapenem-resistant Gram-negative bacteria by species.

Species group	Species	No. of patients	No. of isolates	Patients with intrahospital transmission (%)	No. of intrahospital clusters according to cluster size (no. of patients)					
					Any	2	3	4	5	6
Enterobacterales										
Citrobacter spp.	*Citrobacter braakii*	1	1	0 (0%)	0	0	0	0	0	0
	Citrobacter europaeus	1[a]	2	0 (0%)	0	0	0	0	0	0
	Citrobacter freundii	10	10	0 (0%)	0	0	0	0	0	0
	Citrobacter koseri	4	4	2 (50%)	1	1	0	0	0	0
	Citrobacter meridianamericanus	1	1	0 (0%)	0	0	0	0	0	0
Enterobacter cloacae complex	*Enterobacter asburiae*	6[a]	6	3[b] (50%)	1	0	1	0	0	0
	Enterobacter cloacae	6	6	2 (33%)	1	1	0	0	0	0
	Enterobacter hormaechei	28	30	7 (25%)	3	2	1	0	0	0
	Enterobacter intestinihominis	8	8	2 (25%)	1	1	0	0	0	0
	Enterobacter kobei	7	7	0 (0%)	0	0	0	0	0	0
	Enterobacter ludwigii	1[a]	1	0 (0%)	0	0	0	0	0	0
	Enterobacter pseudoroggenkampii	1	1	0 (0%)	0	0	0	0	0	0
	Enterobacter quasiroggenkampii	1	1	0 (0%)	0	0	0	0	0	0
	Enterobacter roggenkampii	2	2	0 (0%)	0	0	0	0	0	0
Escherichia coli	*Escherichia coli*	22[a]	22	0 (0%)	0	0	0	0	0	0
Klebsiella aerogenes	*Klebsiella aerogenes*	23	24	0 (0%)	0	0	0	0	0	0
Klebsiella oxytoca complex	*Klebsiella michiganensis*	16[a]	16	10 (67%)	3	1	1	0	1	0
	Klebsiella oxytoca	1	1	0 (0%)	0	0	0	0	0	0

Klebsiella pneumoniae complex	*Klebsiella pneumoniae*	41[a]	43	7 (17%)	3	2	1	0	0	0
	Klebsiella quasipneumoniae	1	1	0 (0%)	0	0	0	0	0	0
	Klebsiella variicola	2	2	0 (0%)	0	0	0	0	0	0
Proteus mirabilis	*Proteus mirabilis*	3	3	0 (0%)	0	0	0	0	0	0
Serratia spp.	*Serratia bockelmannii*	1	1	0 (0%)	0	0	0	0	0	0
	Serratia nevei	2	2	2 (100%)	1	1	0	0	0	0
	Serratia sarumanii	2	2	0 (0%)	0	0	0	0	0	0
Nonfermenters										
Acinetobacter spp.	*Acinetobacter baumannii*	8	8	6 (75%)	1	0	0	0	0	1
	Acinetobacter pittii	1	1	0 (0%)	0	0	0	0	0	0
	Acinetobacter soli	1	1	0 (0%)	0	0	0	0	0	0
Chryseobacterium spp.	*Chryseobacterium arthrosphaerae*	3	3	0 (0%)	0	0	0	0	0	0
	Chryseobacterium cucumeris	2	2	0 (0%)	0	0	0	0	0	0
	Chryseobacterium culicis	5	5	0 (0%)	0	0	0	0	0	0
	Chryseobacterium indologenes	6	6	0 (0%)	0	0	0	0	0	0
	Chryseobacterium sp.	1	1	0 (0%)	0	0	0	0	0	0
	Chryseobacterium sp003182335	1	1	0 (0%)	0	0	0	0	0	0
Elizabethkingia spp.	*Elizabethkingia anophelis*	3	3	0 (0%)	0	0	0	0	0	0
	Elizabethkingia bruuniana	1	1	0 (0%)	0	0	0	0	0	0
	Elizabethkingia meningoseptica	2	2	0 (0%)	0	0	0	0	0	0
	Elizabethkingia miricola	2[a]	2	0 (0%)	0	0	0	0	0	0
Metapseudomonas otitidis	*Metapseudomonas otitidis*	6	6	0 (0%)	0	0	0	0	0	0
Pseudomonas aeruginosa	*Pseudomonas aeruginosa*	359[a]	389	52[b] (14%)	21	16	2	1	2	0
	Pseudomonas paraeruginosa	1	2	0 (0%)	0	0	0	0	0	0
Stenotrophomonas spp.	*Stenotrophomonas geniculata*	4	4	0 (0%)	0	0	0	0	0	0
	Stenotrophomonas hibiscicola	1	1	0 (0%)	0	0	0	0	0	0

	Stenotrophomonas maltophilia	46	46	0 (0%)	0	0	0	0	0	0
	Stenotrophomonas muris	10	11	2 (20%)	1	1	0	0	0	0
	Stenotrophomonas pavanii	12[a]	12	0 (0%)	0	0	0	0	0	0
	Stenotrophomonas sepilia	4	4	0 (0%)	0	0	0	0	0	0
	Stenotrophomonas sp031976675	1	1	0 (0%)	0	0	0	0	0	0
	Stenotrophomonas sp913778665	1	1	0 (0%)	0	0	0	0	0	0
Aeromonas spp.	*Aeromonas caviae*	1	1	0 (0%)	0	0	0	0	0	0
	Aeromonas dhakensis	8	8	0 (0%)	0	0	0	0	0	0
	Aeromonas hydrophila	15	15	0 (0%)	0	0	0	0	0	0
	Aeromonas veronii	16	16	0 (0%)	0	0	0	0	0	0
Total		708	750	94 (13%)	37	26	6	1	3	1

[b] One patient was involved in intrahospital transmissions of *Enterobacter asburiae* (cluster size, n=3) and *P. aeruginosa* (cluster size, n=2).

[a] Four patients each had two isolates of different species on the same day: *Citrobacter* spp. and *K. michiganensis*; *E. ludwigii* and *K. michiganensis*; *E. coli* and *K. pneumoniae*; and *E. miricola*. and *S. pavanii*.

Table S2. Sequence types and acquired carbapenemases of 38 genomic clusters.

Species	Cluster ID	No. of isolates	No. of patients involved in intrahospital transmission	No. of patients involved in interhospital transmission	Facility code	Mean days between patient admission and isolate collection	Days between first and last isolate	Sequence type	Acquired carbapenemases
Citrobacter koseri	Citrobacter_koseri-2	2	2	0	I	40.5	166	690	GES-24
Enterobacter asburiae	Enterobacter_asburiae-1	3	3	0	A	16	60	484	IMP-60
Enterobacter cloacae	Enterobacter_cloacae-1	2	2	0	C	37.5	45	53	-
Enterobacter hormaechei	Enterobacter_hormaechei-15	2	2	0	D	25.5	398	133	IMP-1
Enterobacter hormaechei	Enterobacter_hormaechei-22	2	2	0	I	30.5	23	90	IMP-1
Enterobacter hormaechei	Enterobacter_hormaechei-4	3	3	0	B	21.3	634	133	IMP-1
Enterobacter intestinihominis	Enterobacter_intestinihominis-4	2	2	0	B	0.5	48	78	IMP-1
Klebsiella michiganensis	Klebsiella_michiganensis-5	3	3	0	B	40.7	228	43	IMP-1
Klebsiella michiganensis	Klebsiella_michiganensis-8	5	5	0	I	43.4	693	43[a]	IMP-1
Klebsiella michiganensis	Klebsiella_michiganensis-9	2	2	0	I	48.5	6	40	IMP-1
Klebsiella pneumoniae	PPKP2-1	3	3	0	C	51.7	99	15	GES-5
Klebsiella pneumoniae	PPKP294-3	2	2	0	I	1	126	12	IMP-1[b]
Klebsiella pneumoniae	PPKP298-2	2	2	0	B	6.5	54	517	IMP-1
Serratia nevei	Serratia_nevei-1	2	2	0	B	28	328	-	GES-5

Acinetobacter baumannii	PPAC14-1	6	6	0	G	36.8	385	164	IMP-1
Pseudomonas aeruginosa	PPPA11-8	2	2	0	A	11	332	274	-
Pseudomonas aeruginosa	PPPA130-2	3	2	0	B	141.3	215	564	-
Pseudomonas aeruginosa	PPPA16-2	3	2	0	C	95.3	88	348	-
Pseudomonas aeruginosa	PPPA23-1	2	2	0	B	37.5	8	773	-
Pseudomonas aeruginosa	PPPA240-2	2	2	0	C	17	225	2633	-
Pseudomonas aeruginosa	PPPA263-1	2	2	0	C	20	499	3393	-
Pseudomonas aeruginosa	PPPA28-5	6	4	0	A	73.2	232	319	-
Pseudomonas aeruginosa	PPPA30-2	5	5	0	A	42.8	488	313	-
Pseudomonas aeruginosa	PPPA30-4	3	2	0	A	263	132	313	-
Pseudomonas aeruginosa	PPPA305-1	2	2	0	B	3	43	1020	-
Pseudomonas aeruginosa	PPPA30-7	3	3	0	B	92.7	248	313	-
Pseudomonas aeruginosa	PPPA3-1	2	2	0	A	98	417	235	IMP-7
Pseudomonas aeruginosa	PPPA333-1	2	2	0	B	81	157	1420	-
Pseudomonas aeruginosa	PPPA334-1	2	2	0	C	36.5	781	4010	-
Pseudomonas aeruginosa	PPPA337-1	2	2	0	C	1.5	138	550	-
Pseudomonas aeruginosa	PPPA41-3	2	2	0	C	2.5	136	164	-
Pseudomonas aeruginosa	PPPA6-1	2	2	0	E	78.5	27	11	-
Pseudomonas aeruginosa	PPPA61-11	5	5	0	G	34.4	294	186	-
Pseudomonas aeruginosa	PPPA61-8	3	3	0	C	43	292	186	-
Pseudomonas aeruginosa	PPPA73-4	2	2	0	C	140.5	866	606	-
Pseudomonas aeruginosa	PPPA74-1	2	2	0	A	0	461	1027	-
Stenotrophomonas muris	PPSM2-9	2	2	0	F	42	22	905	-
Pseudomonas aeruginosa	PPPA41-2	2	0	2	B, C	77	396	164[c]	-

[a] One isolate was the single-locus variant of ST43.

[b] One isolate appeared negative for IMP-1, but the *bla*$_{IMP-1}$ sequence was split into three parts on separate contigs, which together formed the full gene.

[c] The sequence type of one isolate was untypeable because of the absence of the *ppsA* allele (the other loci were identical to those of ST164).

Table S3. Distribution of AMR genes and mobile genetic elements.

Variable	All			*Enterobacterales*			*P. aeruginosa*		
	Transmitted isolates[a] (n=100)	Non-transmitted isolates (n=650)	P value	Transmitted isolates[a] (n=35)	Non-transmitted isolates (n=162)	P value	Transmitted isolates[a] (n=57)	Non-transmitted isolates (n=334)	P value
Number of AMR genes[b]	5 (5–8)	5 (3–5)	<0.001	8 (7–10)	8 (4–10)	0.454	5 (5-5)	5 (5-5)	0.532
Aminoglycoside	1 (1–2)	1 (1–1)	<0.001	1 (1–2)	1 (0–2)	0.071	1 (1-1)	1 (1-1)	0.546
β-lactam	2 (2–2)	2 (2–2)	<0.001	2 (2–3)	2 (1–3)	0.099	2 (2-2)	2 (2-2)	0.840
Bleomycin	0 (0–0)	0 (0–0)	0.213	0 (0–0)	0 (0–0)	0.133	0 (0-0)	0 (0-0)	NA[c]
Colistin	0 (0–0)	0 (0–0)	0.336	0 (0–0)	0 (0–0)	0.352	0 (0-0)	0 (0-0)	NA[c]
Fosfomycin	1 (0–1)	1 (0–1)	0.102	1 (0–1)	1 (0–1)	0.296	1 (1-1)	1 (1-1)	0.891
Macrolide	0 (0–0)	0 (0–0)	0.246	0 (0–0)	0 (0–0)	0.145	0 (0-0)	0 (0-0)	0.685
Phenicol	1 (1–1)	1 (0–1)	0.003	2 (1–2)	1 (0–2)	0.921	1 (1-1)	1 (1-1)	0.228
Quaternary ammonium	0 (0–1)	0 (0–0)	0.001	1 (1–1)	1 (0–1)	<0.001	0 (0-0)	0 (0-0)	0.082
Quinolone	0 (0–1)	0 (0–0)	0.078	2 (1–3)	2 (1–3)	0.908	0 (0-0)	0 (0-0)	NA[c]
Rifamycin	0 (0–0)	0 (0–0)	0.380	0 (0–0)	0 (0–0)	0.296	0 (0-0)	0 (0-0)	NA[c]
Sulfonamide/trimethoprim	0 (0–1)	0 (0–0)	0.008	1 (0–1)	1 (0–2)	0.352	0 (0-0)	0 (0-0)	0.938
Tetracycline	0 (0–0)	0 (0–0)	0.349	0 (0–1)	0 (0–0.75)	0.489	0 (0-0)	0 (0-0)	0.409
Number of plasmids	1 (0–2)	2 (0–2)	<0.001	3 (2–5)	3 (1–5)	0.109	1 (0-2)	1 (0-1)	0.203
Number of ISs	5 (3–9)	5 (3–7)	0.028	10 (7.5–13)	10 (3–15)	0.690	5 (4-6)	5 (4-6)	0.491
Number of transposons	0 (0–1)	0 (0–1)	0.984	0 (0–1)	0 (0–1)	0.514	0 (0-1)	0 (0-1)	0.156
Integrase genes	37 (37%)	128 (20%)	<0.001	33 (94%)	105 (65%)	<0.001	4	19	0.759

intI1	33 (33%)	118 (18%)	0.001	29 (83%)	95 (59%)	0.007	4	19	0.759
intI2	0 (0%)	3 (0%)	1	0 (0%)	3 (2%)	1	0	0	NA[c]
intI3	4 (4%)	8 (1%)	0.063	4 (11%)	8 (5%)	0.232	0	0	NA[c]

Data are presented as median (IQR) or number (%).

[a] Strains that were involved in intrahospital transmission.

[b] The rows below "Number of AMR genes" represent a breakdown of AMR genes grouped by antimicrobial class, with counts indicating the number of genes associated with each class. Some genes may be counted in more than one antimicrobial class.

[c] P values were not calculated because no positive isolates were observed in either group.

Table S4. Comparison of different definitions of ward/time overlap with other patients.

Characteristic	N (%)		Odds ratio (95% CI)	P value
	Transmitted (n=94)	Non-transmitted (n=614)		
Overlap with other CRGNB-positive patients				
Ward overlap	61 (65%)	297 (48%)	2.0 (1.2–3.1)	0.004
Time overlap	94 (100%)	603 (98%)	Inf (0.4–Inf)	0.376
Ward and time overlap	59 (63%)	291 (47%)	1.9 (1.2–3.0)	0.006
Ward or time overlap	94 (100%)	611 (100%)	Inf (0.1–Inf)	1
Overlap with patients with the same species				
Ward overlap	42 (45%)	163 (27%)	2.2 (1.4–3.5)	<0.001
Time overlap	82 (87%)	420 (68%)	3.2 (1.7–6.1)	<0.001
Ward and time overlap	42 (45%)	158 (26%)	2.3 (1.5–3.7)	<0.001
Ward or time overlap	88 (94%)	457 (74%)	5.0 (2.1–12.1)	<0.001

CI, confidence interval; CRGNB, carbapenem-resistant Gram-negative bacteria; Inf, infinite.

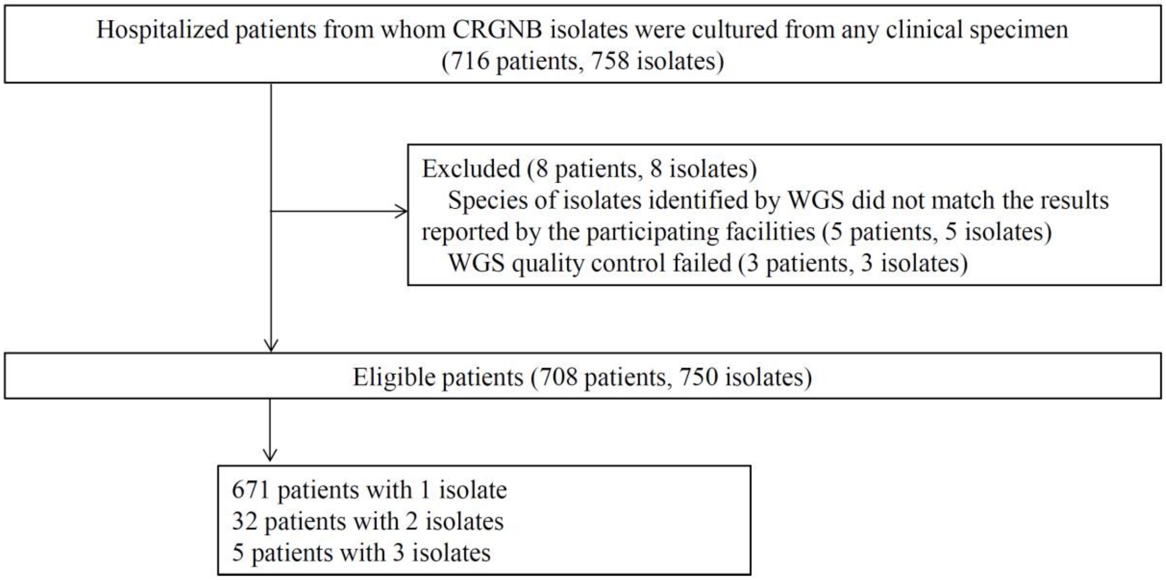

Fig. S1. Flow diagram of patients.

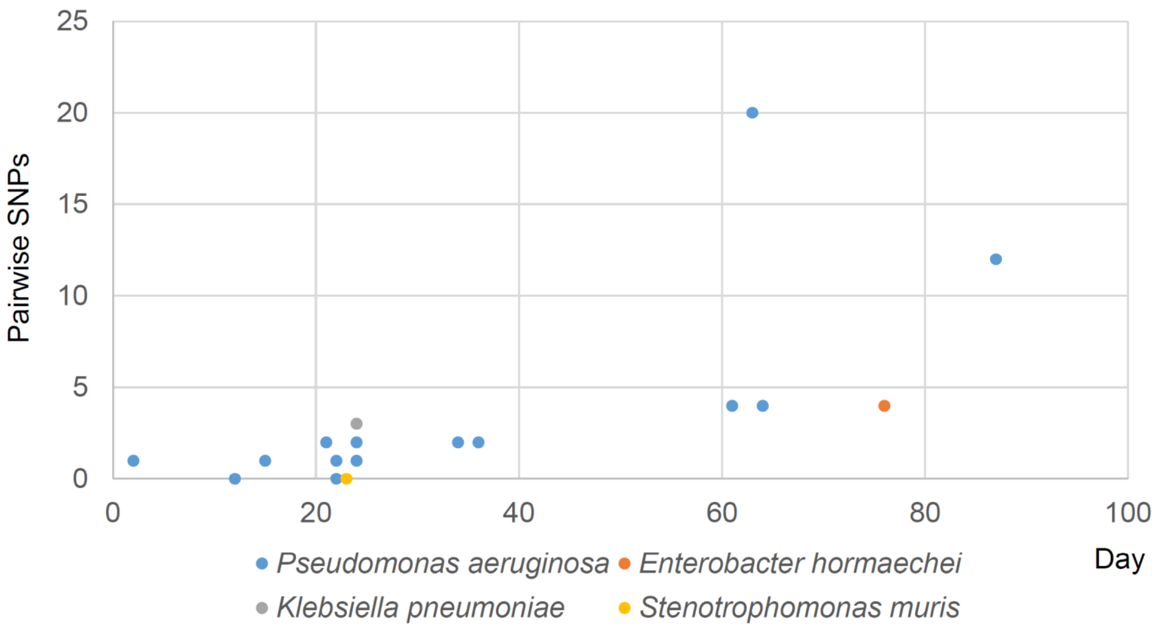

Fig. S2. Pairwise SNP distances and time intervals between isolates of the same species obtained from the same patient 1–90 days apart.

Seventeen patients were included (*P. aeruginosa*, n=14; *E. hormaechei*, n=1; *K. pneumoniae*, n=1; and *S. muris*, n=1). For each patient, only one pair of isolates per patient was analysed. Two patients had 3 *P. aeruginosa* isolates; in these cases, the first and second isolates were used. The SNP distances between the first and subsequent isolates were both 1 (15 and 48 days apart) in one patient and 2 (36 and 61 days apart) in the other. The pairwise SNP distances of *P. aeruginosa* ranged from 0–20 (median, 2; interquartile range, 1–4).

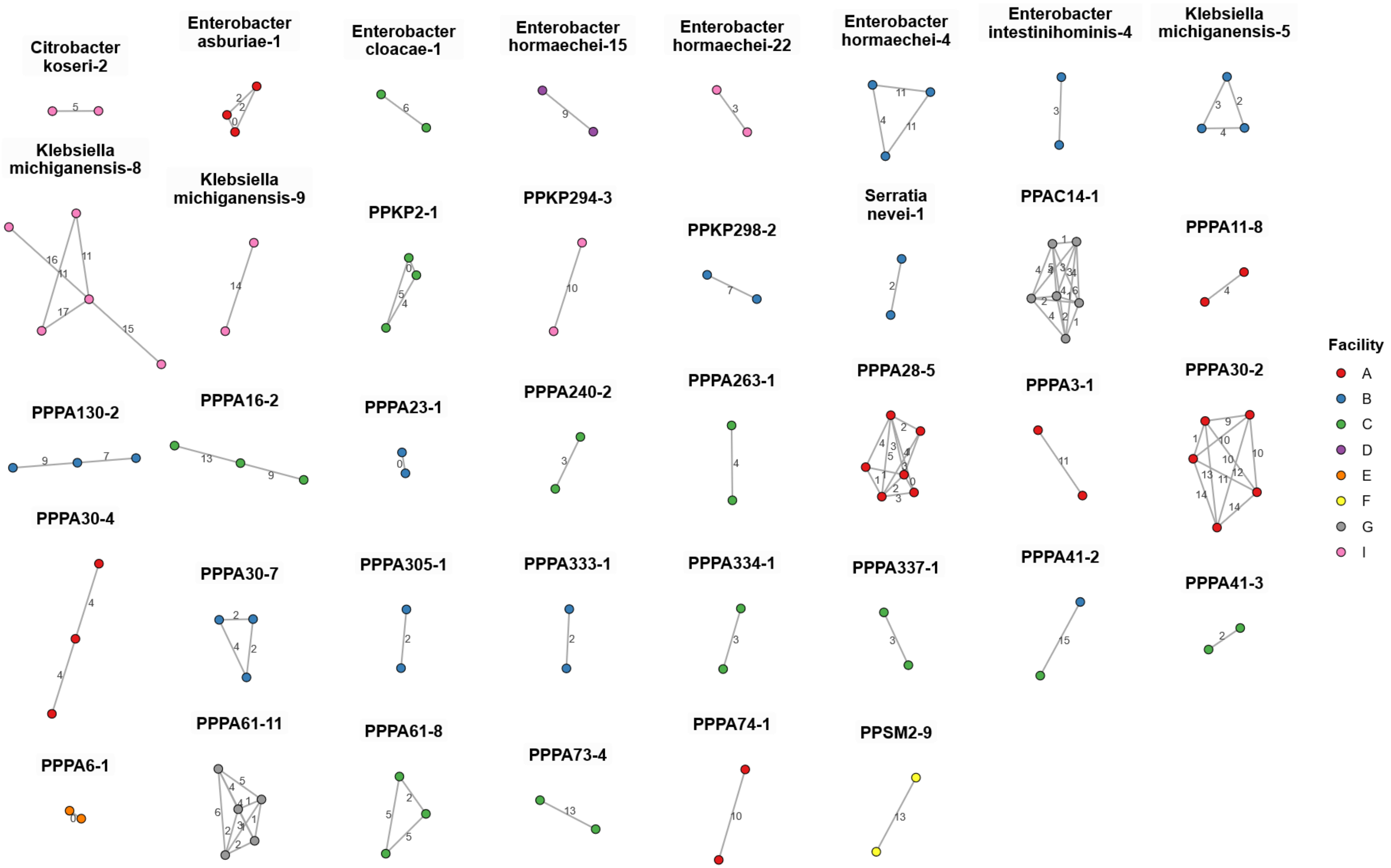

Fig. S3. Network graph showing relationships and SNP distances of isolates within 38 genomic clusters associated with

transmission.

Each circle represents an isolate, coloured according to the facility where the isolate was obtained. A connecting line indicates that the SNP distance of the two isolates is below the species-specific SNP distance threshold. Cluster names correspond to the cluster IDs listed in Table S2, where the species of each cluster are provided. In all the genomic clusters except one (Klebsiella_michiganensis-8), all the pairs of isolates in the same genomic cluster had SNP distances within the species-specific SNP distance threshold. The number on the connecting line indicates the SNP distance; line lengths are not scaled to reflect SNP distance. The median SNP distance within the same genomic cluster was 4 (interquartile range, 2–10). SNP distances were calculated only between isolates obtained from different patients. However, when multiple isolates were available from a single patient, each isolate was included in comparisons with isolates from other patients, and therefore multiple isolates from the same patient may appear in the network.
